# Analysis of resistance to bending of metal electroconductive layers deposited on textile composite substrates in PVD process

**DOI:** 10.1038/s41598-020-65316-2

**Published:** 2020-05-20

**Authors:** Ewa Korzeniewska, Gilbert De Mey, Ryszard Pawlak, Zbigniew Stempień

**Affiliations:** 1 Institute of Electrical Engineering Systems, Lodz University of Technology, ul. Stefanowskiego, 90-924 Lodz, Poland; 2 Department of Electronics and Information Systems Ghent University, Technologiepark Zwijnaarde 126, 9052 Zwijnaarde, Belgium; 3 Institute of Textiles Architecture, Lodz University of Technology, ul. Zeromskiego 116, 90-924 Lodz, Poland

**Keywords:** Electrical and electronic engineering, Characterization and analytical techniques

## Abstract

In the article a description of the behaviour of metallic layers created in the process of physical vacuum deposition on a composite textile substrates during their cyclical bending process is presented. Either the results of experimental research or the theoretical considerations of changes in the structure resistance as a function of the number of fatigue cycles are presented. It was confirmed mathematically that at the beginning of the bending process, in the case of a small number of bends, single cracks appear on the surface of the layer. After exceeding a certain number of bends, the nature of defects on the surface of the layer changes and the dominating mechanism of changes is the widening and elongation of already existing cracks. It has been confirmed mathematically that changes in resistance in these cases depend respectively on the number of bending cycles and next on quadratic value of number of cycles. A correspondence between the mathematical description and experimental results was obtained.

## Introduction

Traditional electronic devices are usually constructed on rigid substrates that, if bent or twisted, have a significant impact on the electronic function of the built-on systems and can even lead to destruction. Change of the substrate to plastic or foil enabling bending or twisting of electrically conductive layers. The elastic electronics is characterized by that feature. Elements of flexible electronics are composed of two layers: a thin passive substrate, e.g. plastic, and from the second functional layer of an electronic element. When a flexible substrate is made of woven fabrics, the term “textile electronics” or textronic is used for those types of systems^1^. Flexible electronics could fill in the gap in some applications such as flexible displays, solar cells. It is used for the production of plastic keyboards as well as electronic labels that protect clothing against theft. The area of elastic electronics usage can also be increased to produce conductive circuits, textile antennas or passive components of electronic circuits. Such systems are used in clothing worn where measurement and control functions are important (temperature, humidity, pressure, kinematic analysis and motion sensors, responses rate monitoring, sensor for cardiac activity, biosensors, etc.)^2–5^. The potential impact of textronics in the field of health protection is significant. Risk assessment and diagnosis will be faster and treatment will be more effective^6,7^.

The production of elastic electronics is possible in spray coating processes using carbon nanotubes, graphene flakes or nanofibers, electroless plating, sputtering, physical vacuum deposition, plasma coating or digital printing^4,8–13^. To fabricate the electroconducting elements on or in textile substrates the varied methods are usually used: the embroidery^14^, dip coating^15^, electrospinning^16^, electroless plating^17,18^, ink-jet or screen printing method^19,20^, thin metallic wires or yarns^3^, metal-coated fibres^21,22^, conductive fibers produced by doping intrinsically conductive polymers^23^, the fibers with metallic core and the outer polymer coating, and even with the liquid metal core^24^, sputtering^25–27^ or chemical and physical vacuum deposition^2,7^. The 3D printing method is also used to direct deposition of electroconductive architectures on textiles. The most common polymer 3D printing technique currently is fused deposition. In this technique, a spool of thermoplastic filament, commonly acrylonitrile butadiene styrene (ABS) or poly(lactic acid) (PLA) is fed to a heating element, which melts the plastic, and forces it out of a nozzle onto a build surface. With the aid of a machine, the plastic is deposited in thin strands that slowly builds up into a 3D shape. For example using that technique Changyong with the group fabricates the tactile sensors^28^ or Zhang *et al*. printed a supercapacitor textile for energy storage^29^.

In flexible and printed electronics 2D structures dominate, e.g. resistors, conductive coils and contacts^2,3,13,15,30,31^. Such structures were sporadically produced using PVD.

Each technique requires different process controlling and monitoring. PVD is one of the environmental friendly method in which no chemical reactions take place, the thin films with high purity can be obtained as well the different shapes of layers could be created using the metals characterized with the best electroconductive properties. Using that method it is relatively easy to create gold, silver or copper layers. It should be also noted that PVD can be preferred in those cases where it is particularly important to achieve the intended shapes and electrical parameters. Due to the usage of the physical vacuum deposition technology, flexible layers of small thickness can be obtained that do not cause additional stiffening of the substrate.

Limitations of the PVD method result from insufficient adhesion of deposited layers resulting from the properties of the substrate material, unstable surface morphology in the case of textile materials. It can result in a lack of electrical conductivity in a larger area. The right choice of layer material can be crucial for the reactive inkjet printing process, were different chemical processes occur^31,32^. In such case, the deposition of gold layers is only acceptable solution. The production of thin electrically conductive gold layers in the PVD process is then economically justified.

Smart textiles are the dominant trend in the textiles development, in particular of wearable electronics^33^. However, the production of intelligent textronic systems is now becoming a reality based on the successful synergy of conventional textile with other fields of science, such as materials science, communication technology, electronics and data processing. The combination of electronics and textile substrates requires the flexibility of the produced circuits and creation of functional thin layers on flexible substrates^34^. The electrical properties depends not only on thickness or the type of deposited metal but also on homogeneity of the layers^35^.

One of the basic conditions of using textronic systems is their mechanical resistance to bending or stretching^36–39^. The fatigue strength properties of thin metallic layers created on polymer substrates (films) have been studied so far due to their usage in flexible electronics^40–44^. There are few literature reports regarding fatigue tests of conductive layers on textile materials^45^. Theoretically, it has been shown that if the layer is well combined with the polymer substrate, the locally generated elongation is suppressed by the substrate and the metal layer can evenly deform far beyond the strength range. However, in the case of delamination, the layer becomes free-standing and breaks at much lower stresses than when it was integrated with the substrate^40^. In most cases described in the literature, thin Cu layers on polymeric substrates are investigated. The dependence of the character of fatigue damages and fatigue life on the thickness of the layer has been demonstrated^41^. The results of research allowed to determine the approximate limit of the layer thickness of about 100 nm, which delimits other mechanisms of fatigue destruction^42^. Above this thickness, as a result of dislocation slip, extrusion and intrusion pairs are created, which initiate microcracks. Below 100 nm, the stresses are so great that cracks can be initiated by structure defects or grain boundaries. To determine the fatigue strength, the measurement of the resistance of the samples is used. In paper^42^, it was shown that the relative change in sample resistance in a long bending cycle can be approximated by two intersecting straight lines, and the intersection defines fatigue strength and corresponds to the number of bending cycles at which the microcracks initiate.

Against the background of the conducted research in the area of the fatigue strength of thin layers, it is important to understand the behaviour of metallic structures created in the process of physical vacuum deposition on a flexible composite substrate. This article attempts to describe the mathematical changes in the resistance of structures depending on the number of bending cycles.

## Materials and Methods

### Materials

In the presented research the electroconductive layers were formed on composite textile substrates during the physical vacuum deposition process. Elastic composite materials commercially named Cordura (Miranda Ltd Poland, surface mass 250 g/m^2^) and PTFE membrane (DEVA F-M, s.r.o., Czech Republic, surface mass 130 g/m^2^) were used as substrates. Cordura is a material made of nylon threads covered in the lamination process with polyurethane film. This material belongs to the group of the most resistant to environmental factors and the propagation of accidental damage. It is used for the production of outdoor equipment and for the production of utility fabrics for special services. The PTFE membrane is made of non-woven fabric on which a thin layer of Teflon film has been stretched. Additionally nanofibers were embedded in the composite production process on the top of PTFE layer. This kind of material is used as water and windproof, primarily in top sports clothing.

Silver and gold with a purity of 99.99% were used as the material vaporized from the tungsten boats used as a thermal resistance source. Metals with the required purity have been provided by the Mennica-Metale Ltd., Poland.

### PVD process

**Figure 1:**
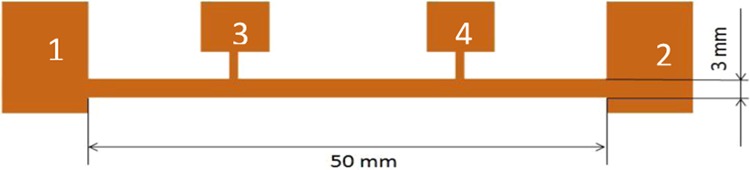
The geometry and structure dimensions of test structure created in the PVD process on textile substrates, 1,2 - current electrodes, 3,4 - voltage electrodes”.

The process of thin electroconductive layers producing was carried out using physical vacuum deposition technology in the chamber of Classic 250 Pfeiffer Vacuum System. The process was carried out after reaching the initial vacuum of 5 × 10^−5^ mbar and lasted 5 minutes. The required geometry of the samples, with the dimensions presented in Fig. 1, was obtained by using previously prepared masks. The mask was created with 3D printer with varied thickness to minimize the shadow effect of masking and to obtain the homogeneous thickness of the thin film.

However, such treatment is not fully profitable due to the shading of the substrate and consequently the heterogeneous thickness of the applied metal. The use of even a thin mask (below 0,5 mm) does not eliminate completely this phenomenon. Therefore, the authors used a thin mask (thickness 0.3 mm) to minimize the mentioned effect. For even better fulfillment of the condition of creating the desired geometry of the structures, the authors suggest the usage of laser ablation in the layer deposited on a large surface in the massless process.

**Figure 2:**
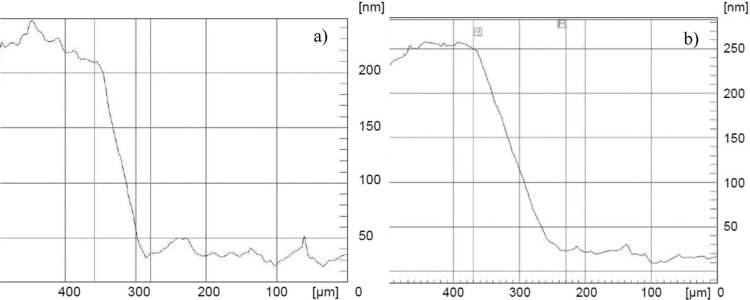
The part of the profile plot of obtained (**a**) gold (**b**) silver thin films.

To determine the thickness of the metal layer, in the same process, a reference glass was placed next to the substrate. Metal layer was also applied on that glass. Next, using the Dektak 3ST profilometer, the thickness of the applied electrically conductive layers was measured (Fig. 2). To explain the phenomena of the dependence of relative change in the sample resistance on the number of bending cycles, the thickness of the layer could be considered as the even and it was 230 nm for Au (Fig. 2a) and 250 nm for Ag (Fig. 2b).

Due to the mechanism of layer creating in the physical vacuum deposition process, it is difficult to obtain the same electrical parameters of the deposited layers each time. The parameters of the metal vapour stream, which mainly result from the temperature and its distribution in the evaporation source (which is not point-like) and the amount of material subjected to evaporation play the decisive role. Even controlling the temperature of the evaporation source by pyrometric measurements will not ensure perfect repeatability.

## Bending Resistance Tests

**Figure 3:**
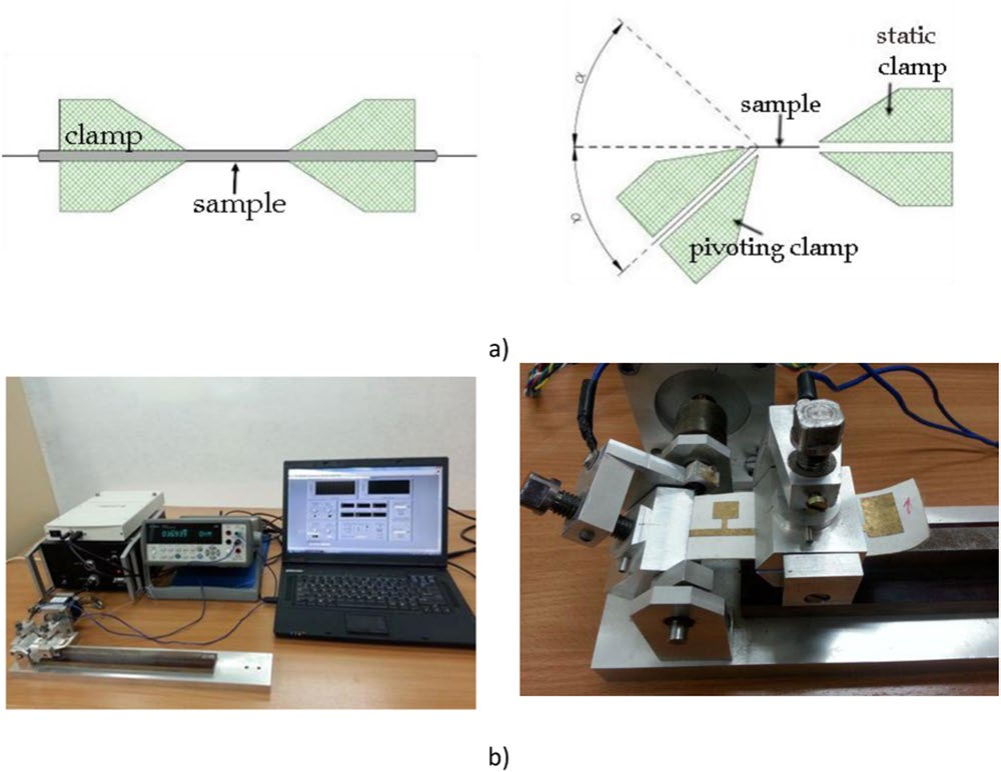
Cyclic bending test: (**a**) measurement idea; (**b**) view of the stand.

Therefore, the presented test results relate to the average values of 20 measurement series. The method of cyclic bending was used to test the mechanical strength of the layers, because it reflects well the real working conditions of textronic systems. The idea of measurement is presented in Fig. 3a^45^. The material sample was fixed in the clamps. One of them was capable of pivoting by an angle of 2α = 90 °. It was important that the axis of rotation of the movable clamp coincides with the edge of the sample fastening. In each cycle, when the deflection angle is 0 °, the sample resistance was measured with a Picotest M3500A ohmmeter. The view of the test bench is shown in Fig. 3b.

The silver layers were subjected to 200 bending cycles. Regardless of the used substrate, after exceeding this number of bends, no continuity of the conducting layer was observed. The different behaviour of resistance during bending cycles was observed in the case of gold layers. Because they are more resistant to fatigue tests, they were subjected to 1500 bending cycles on the Cordura substrate and 50000 ones on the Membrane.

## Experimental Results

**Figure 4:**
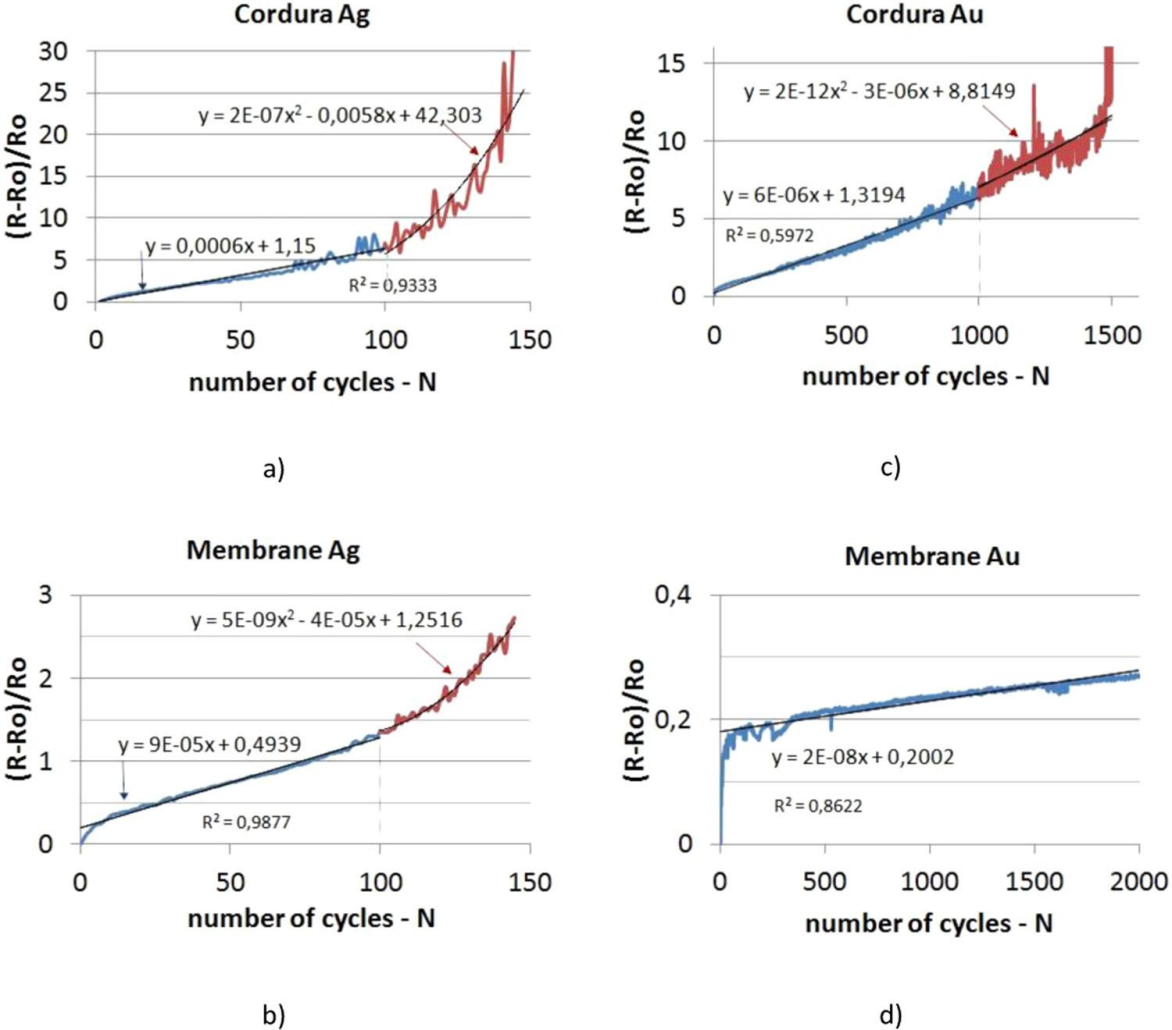
Relative change of resistance of test structures subjected to cyclic bending.

The results of fatigue tests are shown in Fig. 4 in the form of graphs where dependence of relative change of the sample resistance on the number of bending cycles are presented. The fatigue strength of the tested layers should be assessed positively. Taking into account the stringent test conditions (high bending stresses - bending about 90°), the sample resistance increased several times after several hundred or several thousand cycles^45,46^. The presented test results relate to the average values of 20 measurement series.

The nature of the resistance changes for the Ag layers and the Au layers on the Cordura substrate is similar and typical for the behaviour of the metallic layers created on the flexible substrate. The resistance of the Au layers produced on Membrane varied differently. However, in this case it was a very small change of 45% of the initial resistance value up to 50000 cycles. The plot presented in Fig. 4d shows only the linear dependence of relative resistance thin film created on Membrane on number of bending cycles. This kind of phenomena was observed till the end of the conducted test (i.e. 50000 cycles).

Unambiguous criteria (standards) for the evaluation of the results of such tests do not exist, however, according to the authors, it can be assumed that a 10-fold increase in the layer resistance would be in that research acceptable. However, it should be noticed that the assumed criterion should not be define as an acceptance limit in each case. In some applications 10-fold increase in the layer resistance could be accepted but in some other not. For example, when these type of layers will be used as the sensor electrodes and the resistance of active sensor layer will be much higher than the resistance of electrodes, 10-fold increase in the layer (electrodes) resistance could be accepted. However, when these type of layers will be e.g. used as the current collectors in supercapacitor, increase in the layer resistance could limit its performance due to rise of the supercapacitor serial resistance and therefore 10-fold limit would be unacceptable. Because the resistance of the tested layers are characterized by the very small initial value, the authors arbitrary assumed the 10-fold increase in the layer resistance as the good limit value. Using this criterion, the Ag layers on Cordura would survive 120 cycles and 180 cycles on the Membrane. The Au layers created on Cordura would meet this criterion up to 1400 cycles. All changes in resistance at the initial stage were linear (in Fig. 4 they were marked in blue), while the number of cyclic bends increased, the relative change in resistance of the samples changes according to the quadratic function (in Fig. 4 marked in red).

The differences of the silver and gold layers resistance to bending stress results both from the properties of the used substrate and kind of the applied metal. Cordura is a material with less elasticity resulting from both the threads used to make it and from the polyurethane layer (thickness about 100 μm^47^) being the direct substrate for the applied metal layer. Non-woven threads that are a basic component of the Membrane textile composite on which Teflon film has been stretched are thinner and more flexible than the nylon ones which are the part of the Cordura fabric.

Furthermore in the case of the Membrane the metallic layers were characterized by greater adhesion to the substrate due to the presence of microfibers on the surface of the substrate. For this reason, embedded metal layers were associated with this substrate to a much greater extent. The differences in the properties of the substrates explain the behaviour of metallic layers in bending cycles (cf. Figure 4a,c Cordura and Fig. 4b,d Membrane). Research on layer adhesion will be the subject of separate considerations.

According to the authors, the difference in resistance to bending of the tested films results also from the various properties of deposited metals. Gold is the most malleable of all metals so it is also a metal of greater plasticity than silver. Gold hardness is 2.5 on the Mohs scale and the value for silver is a little higher and equals 2.5-3. All features mentioned above contribute to the differences observed between the plots presented in Fig. 4a,b (silver layers) and in Fig. 4c,d (gold layers).

**Figure 5:**
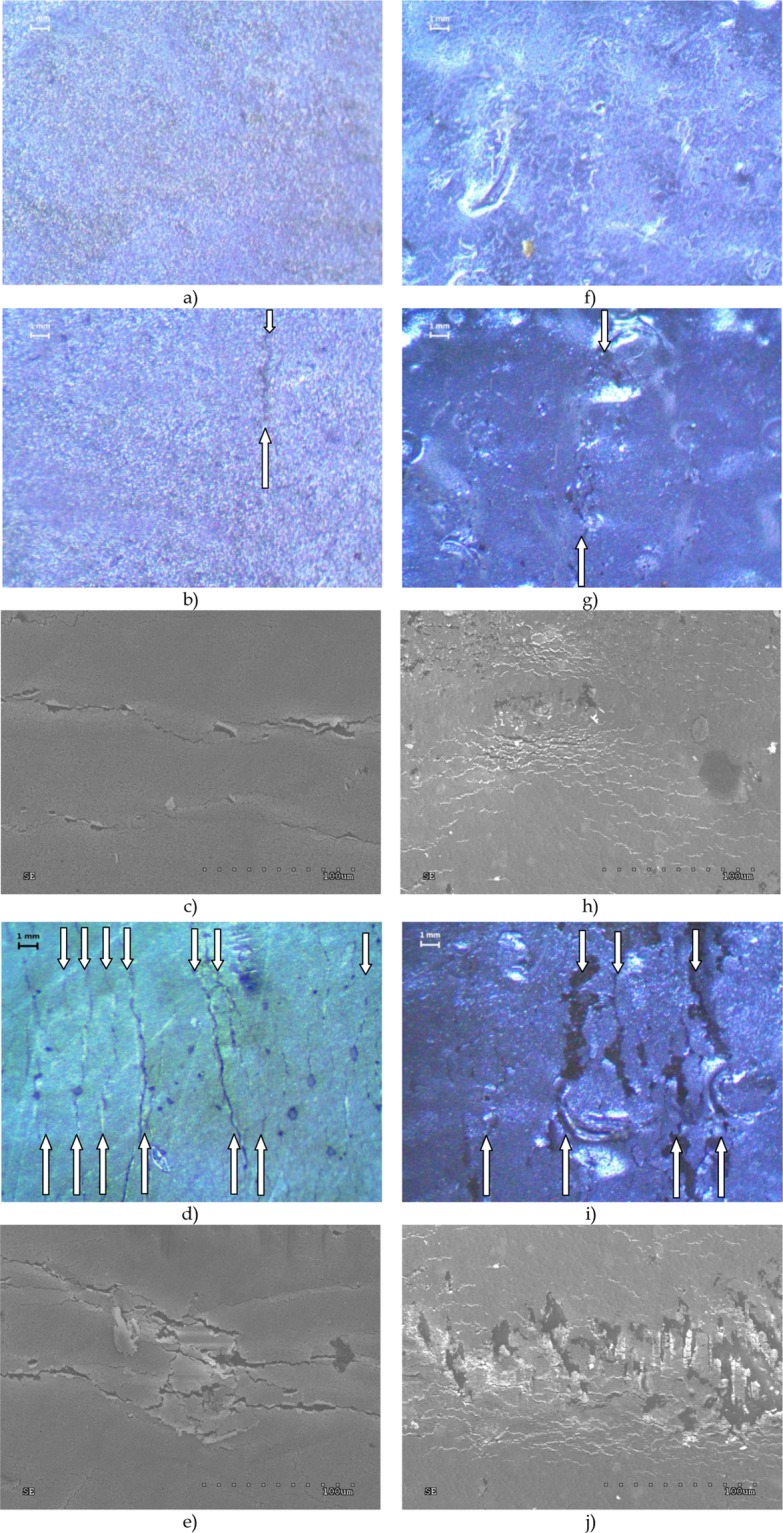
Microscopic images of the surface of samples with thin Ag layer subjected to cyclic bending stress. (**a**) Membrane substrate before bending process - optical microscopy. (**b**) Membrane substrate after 10 bending cycles with single crack - optical microscopy. (**c**) Membrane substrate after 10 bending cycles with single crack - SEM microscopy. (**d**) Membrane substrate after 100 bending cycles with multi cracks - optical microscopy. (**e**) Membrane substrate after 100 bending cycles with multi cracks - SEM microscopy. (**f**) Cordura substrate before bending process - optical microscopy. (**g**) Cordura substrate after 10 bending cycles with single crack - optical microscopy. (**h**) Cordura substrate after 10 bending cycles with single crack - SEM microscopy. (**i**) Cordura substrate after 10000 bending cycles with multi cracks - optical microscopy. (**j**) Cordura substrate after 10000 bending cycles with multi cracks - SEM microscopy.

Observations of the surface of the test samples were carried out using the Optical SZ 630-T optical microscope with a magnification of 60×. The obtained microscopic images are presented in Fig. 5a,b,d,f,g,i. Observations were also carried out using the SEM microscope. The images supplement the information obtained with the optical ones. They were obtained with the Hitachi S-4200 scanning microscope and are shown in Fig. 5c,e,h,j.

The microscopic images presented in Fig. 5 show the structure of the silver layer applied to the Membrane (5a-5e) and Cordura (5f-5j) composite substrates. In pictures b), c) and g), h) single cracks appear in the first phase of the bending process. At a later stage, numerous cracks appear with different widths and distances between each other (Fig. 5d,e,i,j). Their nature varies depending on the used substrate and depends on its flexibility. More minor changes in the wide field are observed on Cordura. The cracks on the Membrane are deeper and less frequent.

## Theoretical Analysis

The theoretical analysis will provide an explanation for the experimentally observed phenomena. For a low number *N* or fatigue cycles, the global electric resistance of the sample varies linearly with *N* or *R ~ N* (Fig. 4). For larger values of *N*, a quadratic relationship *R ~ N*^2^ is observed (Fig. 4). One may wonder whether this is a pure coincidence or is there more behind it? Anyway, the theoretical analysis will prove that a linear or a quadratic relationship can be obtained.

As shown in the photographs (Fig. 5), cracks occur at the moving clamp. To analyse this problem theoretically, one has to calculate the electric resistance of a rectangular sheet having a crack at one contact, which will obviously be the moving contact. Hence, one has to find the potential distribution in the sheet. The latter will be done with a variational technique. Although this is an approximate method, it has proved to be very efficient for the evaluation of resistance and capacitance values^48^. First of all the calculation will be presented in detail for one single crack. Then the results will be extended for multiple but non overlapping cracks.

The purpose of this theoretical analysis is not to provide a complete modelling of the fatigue process. This should require that a lot of parameters have to be taken into account: bending angle, number of cycles, thickness non uniformity, changes of material properties in the neighbourhood of a crack due to the exerted tensile forces. The purpose of the theoretical analysis is just to prove that the electric resistance of the samples changes with the number of bending cycles *N* in a linear and/or quadratic way. Although this is a huge simplification of the fatigue process, it offers the advantage that a simple analytical calculation can be provided. To take into account the number of bending cycles, it is assumed that either a crack becomes longer during each cycle or a new crack is generated.

In a previous published paper^48^, a similar problem was solved using conformal mapping techniques. Although this is an exact analysis, it turned out to be quite complicated. Moreover, a mathematical exact analysis is not necessary taking into account the large variations of the experimental results.

### Variational approach

**Figure 6:**
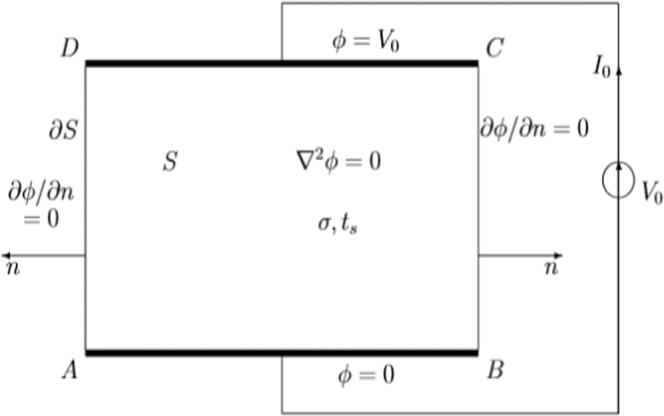
Rectangular geometry of a sample without cracks.

In order to calculate the resistance of a thin layer, one has to evaluate the potential distribution *ϕ*. Once this job is done, one can find the total current *I*_0_ for a given applied voltage V_0_. The resistance is then easily found as *R* = *V*_0_*/I*_0_. In order to find the potential distribution *ϕ(x, y)* one has to solve the Laplace equation:

1 $${\nabla }^{2}\phi =0$$

for the geometry shown in Fig. 6, showing a rectangular shaped resistor without any cracks. At the metallic contacts AB and CD, the potential is known:

2 $$\phi ={V}_{0}\,at\,AB$$

3 $$\phi =0\,at\,CD$$

At the free boundaries BC and DA, i.e. any boundary making contact with a non conducting medium, one has to apply the Neumann boundary condition or the normal derivative should vanish:

4 $$\frac{\partial \phi }{\partial n}=0\,at\,BC\,and\,DA$$

The Eq. (1) with the boundary conditions (2), (3) and (4) can be solved analytically or numerically. Here we will present an approximate analytical method based on the so called variational approach.

The variational method starts with the evaluation of the following functional:

5 $$J=\int {\int }_{S}{[\nabla \phi ]}^{2}dS$$

where *S* denotes the surface of the resistive layer. It has been proved mathematically that the functional *J* reaches its extreme value if one inserts a function *ϕ* in (5) which satisfies the Laplace’ Eq. (1). The variational approach can also be used as an approximation. One inserts a trial function *ϕ*_*trial*_ in (5). *ϕ*_*trial*_ satisfies all the boundary conditions (2), (3) and (4) but not necessarily the Eq. (1). Usually parameters are included in the trial function. One obtains the optimum value of *J* by evaluating the derivative of *J* with respect to these parameters. In such a way one obtains an approximation of the extreme value of *J*.

It has been proved that with a trial function which differs *45%* from the exact solution in some points, the total resistance of a layer was still found with an accuracy of *8%*, although no parameter was included^23^. Although the variational approach is no so widely used, it is the fundamental basis of the finite element method.

A major advantage is that the functional *J* has a physical meaning. Using Gauss theorem we have:

6 $$J=\int {\int }_{S}{[\nabla \phi ]}^{2}dS={\oint }_{\partial S}\phi \frac{\partial \phi }{\partial n}dl={V}_{0}{\int }_{C}^{D}\frac{\partial \phi }{\partial n}dl=\frac{{V}_{0}{I}_{0}}{\sigma {t}_{S}}=\frac{{V}_{0}^{2}}{\sigma {t}_{S}{R}_{0}}\propto \frac{1}{{R}_{0}}$$

where ∂*S* denotes the boundary *ABCDA* of *S*, *σ* is the electric conductivity of the layer and *t*_*S*_ its thickness. V_0_, *σ* and *t*_*S*_ being known, the value of *J* provides us the value of the resistance *R*_0_. An approximate value of *J*, by using a trial potential, will give us then an approximate value of the resistance *R*_0_.

In this paper we are only interested in the change of the resistance due to the bending experiment. *V*_0_, *σ* and *t*_*S*_ do not vary when one or more cracks are formed. If the sheet without any cracks has a resistance *R*_*0*_, which will increase to *R*_*c*_ when a crack is formed, we have using (6):

7 $${J}_{c}-{J}_{0}\propto \frac{1}{{R}_{c}}-\frac{1}{{R}_{0}}$$

where *J*_1_ and *J*_2_ denote the corresponding values of the functional. For small cracks the change of the resistance is small, i.e. *R*_1_ ≈ *R*_2_, hence (7) can be further simplified to:

8 $${J}_{c}-{J}_{0}\propto \frac{1}{{R}_{c}}-\frac{1}{{R}_{0}}=\frac{{R}_{0}-{R}_{c}}{{R}_{0}{R}_{c}}\approx \frac{-{R}_{c}+{R}_{0}}{{R}_{0}^{2}}\propto -{R}_{c}+{R}_{0}$$

In other words, a small variation of the functional value is proportional to the variation of the resistance.

If the bending experiments are carried with a constant current *I*_0_ instead of a constant voltage *V*_0_ the relation (8) hold exactly even for larger variations of the resistance.

### Geometry with a single crack

**Figure 7:**
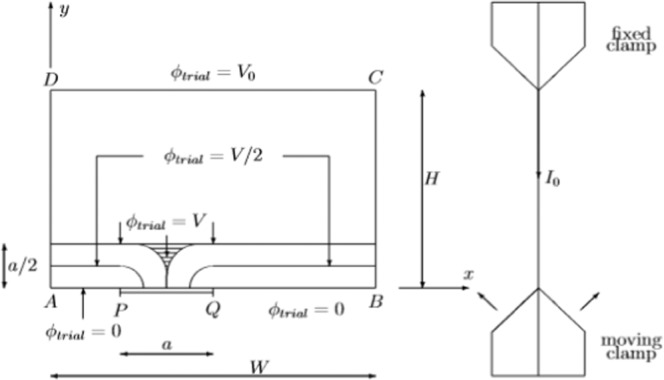
Rectangular geometry of a sample with a single crack PQ at the bottom contact AB.

Consider again a rectangular shaped electric conducting layer with contacts *AB* and *CD*. Due to the moving clamp a crack *PQ* has been created (Fig. 7). As a consequence the electric contact is limited to *AP* and *QB*. The crack *PQ* does not supply any current and has to be treated mathematically as a free boundary.

The trial function is displayed graphically in Fig. 7. The value *V* has been introduced as a parameter. For *a/2* < *y* < *H*, the potential *ϕ*_*trial*_ varies linearly between *V* and *V*_0_. For 0 < *y* < *a/2* the potential *ϕ*_*trial*_ also varies linearly between 0 and *V*, but only above *AP* and *QB*, i.e. where there is no crack. Above the cracks, the equipotential lines of the trial function *ϕ*_*trial*_ are bended as quarter circles. In the striped zone the potential is just constant: *ϕ*_*trial*_ *= V*. One can easily verify that the trial function satisfy all boundary conditions. *∂ϕ*_*trial*_*/∂y = 0* on the crack *PQ*, *∂ϕ*_*trial*_ */∂x = 0* on both sides *BC* and *DA*, *ϕ*_*trial*_ = 0 at the contacts *AP* and *QB* or the non cracked zone. At last ϕ_trial_ = V_0_ at the top contact *CD*.

Inserting the trial function *ϕ*_*trial*_ in the functional (5) reads:

9 $${J}_{1}=\int {\int }_{S}{(\nabla {\phi }_{trial})}^{2}dxdy={V}^{2}\left[\frac{2W}{a}-\frac{4-\pi }{4}\right]+{({V}_{0}-V)}^{2}\frac{W}{H-\frac{a}{2}}$$

The above expression still contains an unknown parameter: *V*. To find this value, we have to look for the optimum value of the functional *J* by evaluating *∂J*_1_*/∂V =* 0. One obtains:

10 $$V=\frac{\frac{{V}_{0}W}{H-a/2}}{\frac{2W}{a}-\frac{4-\pi }{4}+\frac{W}{H-a/2}}$$

Inserting this value in (9) and taking into account that *a* is a small value, gives us the optimum value for the functional *J*_*1,opt*_:

11 $${J}_{1,opt}=-\frac{{V}_{0}^{2}}{{H}^{2}}\left[\frac{4-\pi }{4}+{\left(\frac{4-\pi }{4}\right)}^{2}\right]\frac{{a}^{2}}{4}+\frac{{}^{}{V}_{0}^{2}W}{H}$$

The last term of (11) is the value of *J*_*opt*_ for *a* = *0* or *J*_*opt*_*(a* = *0)* = *V*_*0*_^2^*W/H*. Hence the variation of the functional which, according to (8) is also the change of the resistance value due to a single crack is given by:

12 $${J}_{1,opt}-{J}_{1,opt}(a=0)=-\frac{{V}_{0}^{2}}{{H}^{2}}\left[\frac{4-\pi }{4}+{\left(\frac{4-\pi }{4}\right)}^{2}\right]\frac{{a}^{2}}{4}\propto -{R}_{c}+{R}_{0}$$

Most important conclusion is that the change of the resistance is proportional to the square of the crack’s length *a*.

### Multiple cracks

**Figure 8:**
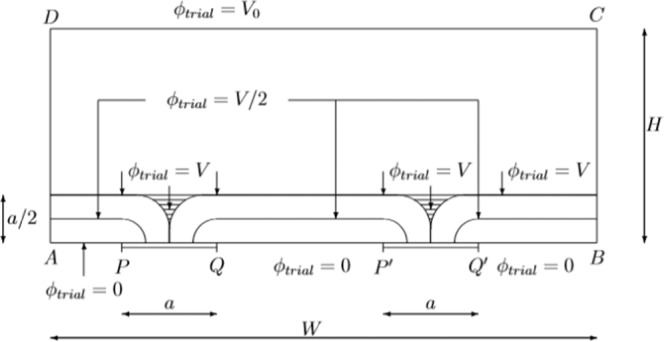
Rectangular geometry of a sample with two cracks PQ and P’Q’at the bottom contact AB.

We consider now the case that *n* cracks, each of them having the same length *a*, are existing along the moving contact *AB*. In Fig. 5 the particular case *n* = 2 has been displayed in Fig. 8.

The trial function *ϕ*_*trial*_ to be used now, is drawn schematically in Fig. 8 as well. The calculation is almost identical to the one of the preceding section, so that the mathematical details can be skipped here. For the geometry of Fig. 8, the functional is now called J_2_:

13 $${J}_{2,opt}-{J}_{2,opt}(a=0)=n\frac{{V}_{0}^{2}}{{H}^{2}}\left[\frac{4-\pi }{4}+{\left(\frac{4-\pi }{4}\right)}^{2}\right]\frac{{a}^{2}}{4}\propto -{R}_{c}+{R}_{0}$$

The conclusion is obvious, the change of the resistance is proportional to the number of cracks *n*.

From the theory outlined so far, we come up with the knowledge that a new crack is not the same as a crack which is doubling its length. The first one gives a change in resistance which varies linearly with the number of cracks (~*n*) whereas the second one gives rise to a quadratic behaviour (~*n*^2^).

Note that this theoretical analysis is only valid as long as the lengths of the cracks are sufficiently small, at least much smaller than the width *W* of the contacts. It must be also remarked that the purpose of this theoretical analysis is not to get a mathematical expression which can be fitted accurately to the experimental data. We just wanted to prove the two relations ~*n* and ~*n*^2^.

## Discussion

It has been proved theoretically that if the number of cracks *n* is growing, the change of the resistance is proportional to *n*. From the experimental curves (Fig. 4), a linear relationship is observed for small values of *N*, i.e. the number of fatigue cycles. Hence we may conclude that in the beginning (small *N*) cracks are created during each cycle. The more cycles, the more cracks.

The theory also reveals that if the number of cracks remains constant but the cracks are growing in length, a quadratic relationship is found (~*n*^*2*^). The experiments (Fig. 4) show a quadratic relationship with the number of fatigue cycles *N*, at least for larger values of *N*. One may conclude that after a certain number of fatigue cycles, existing cracks become longer and new cracks are no longer created.

By comparing the theory with the experiments, the conclusion is that in the beginning of the fatigue experiment, the creation of cracks is the dominant mechanism. After some fatigue cycles the dominant mechanism is the elongation of the previously created cracks.

The proposed theoretical description allows better understanding of the behaviour of the tested layers during bending cyclic loads, which has a direct impact on the operational fatigue strength of such layers. In some applications, e.g. as the conductive electrodes or tracks, the change of the resistance up to 10 times is acceptable because the initial resistance is low. Such fatigue strength is demonstrated by the layers in the area where the change in resistance is linearly dependent on the number of cycles.

Additionally, it should be noticed that the presented theoretical model was developed under the assumptions of isotropic properties of textile composites. These isotropic properties can be assumed for the non-woven fabric coated with Teflon. In case of structures based on woven fabric, the interface layers were isotropic, however the raw woven fabrics typically are anisotropic.

## Conclusion

On the basis of the experiment, a mathematical description was created to understand the nature of changes in resistance of metallic thin layers created on the composite textile substrates depending on bending stress and prediction of their strength.

During the research, two ranges of load (number of bending cycles) were distinguished in which the strength behaviour of the layer is of a different nature. In the initial phase of tests, the resistance of structures depends linearly on the number of bends. This is related to the appearance of new cracks on the surface of the layer. In the next part of the research, when new cracks no longer arise, existing defects widen and lengthen. In this case, the change in resistance depends on the square of the number of bends. The presented description was confirmed in microscopic observations. This method in combination with microscopic images describes the behaviour of the layer in the fatigue process.

The results of the conducted experiments confirm the validity of the model. Further evaluation of the model could relate to its use for layers applied with another technique.

The purpose of further research is an attempt to modify the technology of creating of metallic structures on composite substrates to broaden the linear range of their strength, thereby increasing the load range of the created layers.
